# Effects of β-glucan with vitamin E supplementation on the growth performance, blood profiles, immune response, fecal microbiota, fecal score, and nutrient digestibility in weaning pigs

**DOI:** 10.5713/ab.22.0311

**Published:** 2022-11-14

**Authors:** Tae Wook Goh, Hong Jun Kim, Kunyong Moon, Cheon Soo Kim, Yoo Yong Kim

**Affiliations:** 1 Department of Agricultural Biotechnology and Research Institute of Agriculture and Life Sciences, Seoul National University, Seoul 08826, Korea

**Keywords:** Fecal Score, β-Glucan, Growth Performance, Intestinal Microbiota, Vitamin E, Weaning Pigs

## Abstract

**Objective:**

This study was conducted to evaluate effects of β-glucan with vitamin E supplementation on the growth performance, blood profiles, immune response, fecal microbiota, fecal score, and nutrient digestibility in weaning pigs.

**Methods:**

A total of 200 weaning pigs with an average body weight (BW) of 7.64±0.741 kg were allotted to five treatment groups and were divided based on sex and initial BW in four replicates with ten pigs per pen in a randomized complete block design. The experimental diets included a corn-soybean meal-based basal diet with or without 0.1% or 0.2% β-glucan and 0.02% vitamin E. The pigs were fed the diets for 6 weeks. A total of 15 barrows were used to evaluate the nutrient digestibility by the total collection method. The BW and feed intake were measured at the end of each phase. Blood samples were collected at the end of each phase, and fecal samples were collected at the end of the experiment.

**Results:**

The addition of β-glucan with vitamin E to weaning pig feed increased BW, average daily gain, and average daily feed intake. A significant decrease in yeast and mold and Proteobacteria and a tendency for Lactobacillus to increase compared to the control was shown when 0.1% β-glucan and 0.02% vitamin E were added. The fecal score in weaning pigs was lower in the treatments supplemented with 0.1% or 0.2% β-glucan and 0.02% vitamin E compared to the control. In addition, vitamin E was better supplied to weaning pigs by increasing the concentration of α-tocopherol in the blood of weaning pigs when 0.02% vitamin E was supplemented. However, there was no significant difference in either the immune response or nutrient digestibility.

**Conclusion:**

Inclusion of 0.1% β-Glucan with 0.02% vitamin E in a weaning pig’s diet were beneficial to the growth performance of weaning pigs by improving intestinal microbiota and reducing the incidence of diarrhea.

## INTRODUCTION

Antibiotics have been widely used in feed for weaning pigs to improve feed efficiency, promote growth, and reduce diseases. However, the European Union and Korea banned the use of antibiotics as feed additives for growth promotion in 2006 and 2011, respectively [1]. For this reason, research on various antibiotic substitutes, such as plant extracts, probiotics, and β-glucan (BG), has been actively conducted.

β-Glucan is a complex carbohydrate extracted from mold, grains, and the cell walls of yeast. Glucans with β-1,3 and β-1,6 glycosidic bonds are major structural components of yeast and fungal cell walls, where these bonds play a role in disease defense and growth promotion [2]. β-Glucan can stimulate a series of pathways that activate the immune system and enhance both innate and adaptive immune responses [3]. β-Glucan has antitumor and antibacterial activities by enhancing host immune function [4]. It has a beneficial effect on the growth of weaning pigs because it induces a specific immune response and increases nonspecific immunity and tolerance to oral antigens as an immune modulator [5].

Vitamin E, which is a very important nutrient for pigs, is known to enhance immunity through antioxidant action. In particular, vitamin E (VE), which acts as an antioxidant at the cellular level, has a structural function and performs various functions related to reproduction [6]. In addition, VE improves the immune response to antigens by stimulating the production of lymphocytes [7]. Additionally, it is an important component of all membranes found in cells, including plasma, mitochondria, and nuclear membranes [8].

There are many previous studies on the effects of BG and VE individually on weaning pigs, but there is insufficient evidence to verify the synergistic effect of BG and VE on weaning pigs. Thus, it was hypothesized that the synergistic effects of BG and VE could improve immunity, leading to an increase in growth performance in weanling pigs. Therefore, this study was conducted to evaluate the effects of BG with VE on the growth performance, blood profiles, immune response, fecal microbiota, fecal score, and nutrient digestibility of weaning pigs.

## MATERIALS AND METHODS

All experimental procedures involving animals were conducted in accordance with the Animal Experimental Guidelines provided by the Seoul National University Institutional Animal Care and Use Committee (SNUIACUC; SNU-200209-2)

### Experimental animals and housing environment

A total of 200 weaning pigs ([Yorkshire×Landrace]×Duroc) with an initial body weight (BW) of 7.64±0.741 kg were allotted to one of five treatments considering sex and initial BW in four replicates with ten pigs per pen in a randomized complete block design. Pigs were randomly assigned to their respective treatments by the Experimental Animal Allotment Program (EAAP) [9]. Pigs were housed in an environmentally controlled facility. The pens had fully concrete floors (1.54×1.96 m^2^). Feed and water were provided *ad libitum* through a feeder and a nipple during whole experimental periods. The temperature was kept at 30°C during the first 7 days and lowered 1°C every week. The experimental period was 6 weeks (phase I, 0 to 3 weeks; phase II, 3 to 6 weeks). Body weight and feed intake were measured at the end of each phase to calculate the average daily gain (ADG), average daily feed intake (ADFI), and gain:feed ratio (G:F ratio).

In addition, feed given to all piglets was recorded each day, and feed waste in the feeder was recorded at the end of each phase.

### Experimental design and diet

Dietary treatments included i) CON (corn–soybean meal [SBM]-based diet), ii) LB (corn-SBM-based diet + BG 0.1%), iii) LBE (corn-SBM-based diet + BG 0.1% + VE 0.02%), iv) HB (corn-SBM-based diet + BG 0.2%), and v) HBE (corn-SBM-based diet + BG 0.2% + VE 0.02%). A corn-SBM-based diet was used as feed in this experiment, and all nutrients in the experimental diet except crude protein (CP) met or exceeded the nutrient requirements of the National Research Council (NRC) [10] for weaning pigs. The CP was set to 6.25 times more than the standard total nitrogen in the requirement of NRC [10] to calculate CP requirements. In the present study, BG and VE products were provided by E&T Company (E&T CO., Ltd, Daejeon, Korea). β-Glucan consisted of (1,3)–(1,6)-β-D-glucan and mannan. Vitamin E was in the form of VE-acetate. In the case of VE, 65 IU/kg was present in the vitamin premix, and 110 IU/kg of VE was additionally supplemented to the LBE and HBE treatments. All nutrient contents in the feed were formulated equally, and the formula and chemical composition of the experimental diet are presented in Table 1 and 2. The CP content of phase I in the weaning pig feed was 20.56%, the lysine content was 1.35%, the methionine content was 0.39%, the cysteine content was 0.35%, the threonine content was 0.79%, the tryptophan content was 0.22%, the calcium (Ca) content was 0.80%, and the total phosphorus (P) content was 0.65%. The CP content of phase II in the weaning pig feed was 18.88%, the lysine content was 1.23%, the methionine content was 0.36%, the cysteine content was 0.32%, the threonine content was 0.73%, the tryptophan content was 0.20%, the Ca content was 0.70%, and the total P content was 0.60%.

### Blood profiles and immune response

Blood samples were taken from the jugular vein of three pigs near the average BW in each treatment after 3 hours of fasting on the initial day and at the end of each phase to measure VE, selenium (Se), tumor necrosis factor-α (TNF-α), interleukin-6 (IL-6), and lymphocytes. All blood samples were collected in serum tubes (SST II Advance; BD Vacutainer, Becton Dickinson, Plymouth, UK) and centrifuged at 1,957×g and 4°C for 15 min (5810R; Eppendorf, centrifuge 5810R, Hamburg, Germany). Subsequently, the supernatant was separated in a microtube (AXYGEN. INC, Union City, CA, USA) and the samples of VE, IL-6, and TNF-α were stored at −20°C, while the samples of Se and lymphocytes were stored at 4°C for analysis. Each measurement was conducted using the following analysis machines and techniques: Se (ICP–MS; Perkin Elmer, Rodgau, Germany), VE (HPLC-UVD; PerkinElmer, Milford, MA, USA), lymphocytes (Flow cytometry, automatic blood analyzer; Sysmex, Hyogo, Japan), TNF-α (Fluorescent, Luminex; Millipore, Austin, TX, USA), and IL-6 (Fluorescent, Luminex; Millipore, USA).

### Fecal microbiota

Measurements of microorganisms in feces were performed at the end of the experiment. Fecal samples were collected based on the BW of the pigs in the treatments, transported to the laboratory on ice, and stored in a −80°C freezer until further analysis. One milliliter of the pretreated sample was diluted 10-fold in steps in 9 mL of sterile 0.1% peptone water, and 1 mL of sample was taken at each dilution concentration and dispensed in 3 M dry film medium to analyze aerobic bacteria, coliform, *E. coli*, lactic acid bacteria, yeast, and mold. Subsequent triplicate spread plating was performed on Petrifilm aerobic plate count (APC) plates, Petrifilm coliform count plates, and Petrifilm yeast and mold count (YM) plates according to the manufacturer’s instructions. APC and coliform plates were incubated aerobically at 37°C for 24 h, and yeast and mold plates were incubated aerobically at 25°C for 72 h in an aerobic incubation chamber. Counts were recorded as colony forming units per gram (CFU/g). In addition, fecal sample deoxyribonucleic acid (DNA) was extracted for metagenomic analysis using the DNeasy PowerSoil Pro kit (Qiagen, Hilden, Germany) according to the manufacturer’s protocol for comparison with the culturomic approach. All bacteria isolated through 16S rRNA sequencing were identified and classified, and the microbial composition of fecal samples was analyzed through metagenomics using next-generation sequencing (NGS) technology.

### Fecal score

Observations of fecal scores were made every day at 08:00 throughout the feeding trial (35 days). Data were recorded by one trained researcher for each pen. Fecal scores were given according to the condition of feces (0 = normal feces; 1 = moist feces; 2 = mild feces; 3 = watery diarrhea) [11]. Slightly wet feces on the rump area were used to designate contaminated piglets. After recording the data, we cleaned away the feces by wiping off the fecal areas or the pig’s butt, preparing for a new measurement the next day.

### Nutrient digestibility

A total of 15 crossbred barrows, averaging 12.48±0.37 kg BW, were allotted to individual metabolic crates (40×80×90 cm) in a completely randomized design with three replicates to evaluate nutrient digestibility and nitrogen retention. The total collection method was used to determine the apparent total tract digestibility of dry matter (DM), CP, crude ash, and crude fat [12]. After a five-day adaptation period, there was a five-day collection period. To determine the first and last day of collection, 8 g of ferric oxide and chromium oxide were added to the first and last experimental diets as selection markers. During the experimental period, all pigs were fed the phase II diets twice per day, at 07:00 and 19:00, which provided three times the maintenance energy [13], and water was provided *ad libitum*. Collection of feces was started when ferric oxide appeared in the feces and was maintained until the appearance of chromium oxide in the feces. Urine samples were collected during the collection period in plastic containers containing 50 mL of 4 N H_2_SO_4_ to prevent evaporation of nitrogen prior to nitrogen retention analysis. Fecal and urinary samples were stored at −20°C until the end of the collection period, and the feces were dried in a drying oven at 60°C for 72 h and then ground to 1 mm in a Wiley mill (CT 193 Cyclotec; FOSS, Höganäs, Sweden) for chemical analysis, including moisture, CP, crude fat, and crude ash contents, by the Association of Official Analytical Chemists (AOAC) methods [14].

### Chemical analysis

The diets and feces were ground by a Wiley mill (CT 193 Cyclotec; FOSS, Sweden) and then analyzed for DM (procedure 967.03; [14]), ash (procedure 923.03; [14]), and ether extract (procedure 920.39; [14]). The nitrogen content was analyzed using the Kjeldahl procedure with Kjeltec (KjeltecTM 2200; Foss Tecator, Sweden) and by calculating the CP content (nitrogen×6.25; procedure 981.10; [14]).

### Statistical analysis

All obtained data were processed by Excel 2010 first, and then analyzed by one-way ANOVA procedure using Statistical Analysis System 9.4 TS1M7 (SAS Inst. Inc., Cary, NC, USA). Each pen was used as the experimental unit for growth performance and fecal score, while individual pigs were used as the experimental unit for fecal microbiota, blood profiles, and nutrient digestibility. The orthogonal polynomial contrasts were used to determine the effects of diet (BG and VE against the control), BG, VE, as well as the interaction between BG and VE. Data were presented as means and their pooled standard errors. The differences were considered as statistically significant when p<0.05, while 0.05≤p<0.10 was considered to indicate a trend in the data.

## RESULTS AND DISCUSSION

### Growth performance

The effects of BG with VE supplementation in the weaning pig diet on growth performance are shown in Table 3. As a result, BW at week six, ADG for the entire period of the experiment, and ADFI for phase II (3 to 6 weeks) were significantly higher in all treatment groups to which BG or VE was added compared to the control group (Diet, p<0.05). In addition, the treatment groups supplemented with 0.2% BG compared to those supplemented with 0.1% BG showed significantly higher in BW at week six (BG; p<0.01), and a higher trend in ADG at phase II and the overall experimental period (BG; p = 0.09, p = 0.08). The addition of 0.02% VE significantly increased BW at week six and ADG in phase II (VE; p<0.01). The HB and HBE treatments with 0.2% BG had significantly higher ADFI than the treatments with 0.1% BG in phase II and entire experimental period (BG; p<0.05). Adding 0.02% VE showed a significantly higher G:F ratio in phase II than treatments without VE (p<0.05).

Park et al [15] observed that supplementation with BG linearly increased ADG in phase I (0 to 2 weeks) and the entire period (6 weeks) and linearly decreased the feed conversion ratio in phase I (0 to 2 weeks), phase II (2 to 6 weeks), and the entire period (6 weeks) when they compared supplementation of BG by level (0%/0.1%/0.2%/0.4%) in the weaning pig diet with the treatment supplemented with 0.003% antibiotic Tiamulin. Luo et al [16] reported that supplementing 0.01% BG in the weaning pig diet increased ADG linearly and quadratically (p<0.05) during the entire experimental period (28 days) when BG by level (0%/0.0025%/0.005%/0.01%/0.02%) was supplemented. Pigs fed 0.005% BG had significantly higher ADG (p<0.05) during the whole experimental period (28 days) and increased ADFI (p<0.05) during 0 to 28 days and 28 to 35 days when treatments with 0.005% BG were compared with the control [17]. Pigs supplemented with 0.1% each BG from mulberry leaves and curcuma had significantly higher ADGs and G:F ratios than the control (p<0.05) in Lee et al [18] experiment during phase I (1 to 14 days). On the other hand, Zhou et al [19] reported that there was no significant difference in the growth performance of weaning pigs when 0.01% BG was fed to weaning pigs challenged with lipopolysaccharide.

Most previous studies reported that the addition of BG to weaning pig feed had a positive effect on growth performance, but the exact mechanism for improvement in growth performance was not elucidated [20].

However, BG, which is used as a broad-spectrum immune enhancer, has contributed to increasing the growth performance of animals such as swine by strengthening the intestinal mucosa of piglets and improving the intestinal environment when it is supplemented in the weaning pig diet [18,21]. The ADFI of weaning pigs was higher than that of the control because the immune and health status of weaning pigs were improved considering the results of previous studies.

Therefore, as a result of the present experiment, the addi tion of BG and VE to weaning pig feed increased BW, ADG, and ADFI. Additionally, supplementation with 0.2% BG and 0.02% VE had a positive effect on the growth performance of weaning pigs.

### Blood profiles and immune response

The effects of BG with VE supplementation in the weaning pig diet on blood profiles and immune response are shown in Table 4. There was an increasing trend in VE concentration in the blood profiles in groups supplemented with 0.02% VE (p = 0.08). However, there was no significant difference in the blood concentrations of selenium, TNF-α, IL-6, and lymphocytes (p>0.05).

Moreira and Mahan [22] reported that there was a significant increase in the average level of VE between days 7 and 35 in treatments supplemented with VE compared to the control without VE when VE was added by level (0 IU/20 IU/40 IU/60 IU) in a weaning pig diet (p<0.05). In addition, supplementation with 250 IU VE increased the concentration of VE significantly on days 42 and 68 compared to the treatment supplemented with 40 IU in the study of Rey et al [21] (p<0.01).

Various factors influence the VE status of pigs before and after weaning. Neonatal piglets are born with a low α-tocopherol concentration in their tissues [6]. Diarrhea after weaning lowers serum α-tocopherol concentration and worsens VE absorption [23]. In addition, VE deficiency occurs most frequently in weaning pigs during the first few weeks after weaning, as postweaning serum VE concentrations decrease due to low feed intake and increased stress.

In the current experiment, the treatments with 0.02% VE added to the weaning pig feed showed an increasing trend in VE concentration at week three compared to the treatments without VE. As in the previous studies of Rey et al [21] and Moreira and Mahan [22], the concentration of VE in the blood tended to increase with additional VE supply. This means that VE is being easily delivered into the piglets according to the additional VE supply in the weaning pig feed. It can also be expected that VE will have a positive effect on improving the antioxidant status of weaning pigs and increasing the immune response through enhanced cell protection.

In the current experiment, the addition of 0.02% VE in the diet of weaning pigs had a positive effect on the VE concentration in weaning pigs.

### Fecal microbiota

**Figure 1:**
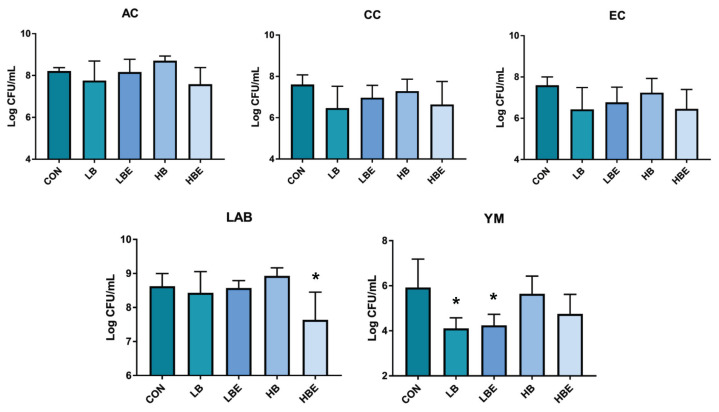
Fecal microbiota according to the addition of β-glucan with vitamin E in weaning pig feed (comparison of all treatments; quantitative analysis). Counts were recorded as colony forming units per gram (CFU/g). Significant differences in the figure are expressed as *, and differences were considered significant at p<0.05. CON, corn-SBM based diet; LB, corn-SBM based diet+0.1% β-glucan; LBE, corn-SBM based diet+0.1% β-glucan+0.02% vitamin E; HB, corn-SBM based diet+0.2% β-glucan; HBE, corn-SBM based diet+0.2% β-glucan+0.02% vitamin E. AC, aerobic count; CC, coliform count; EC, *E.coli*/coliform count; LAB, lactic acid bacteria count; YM, yeast and mold count.

**Figure 2:**
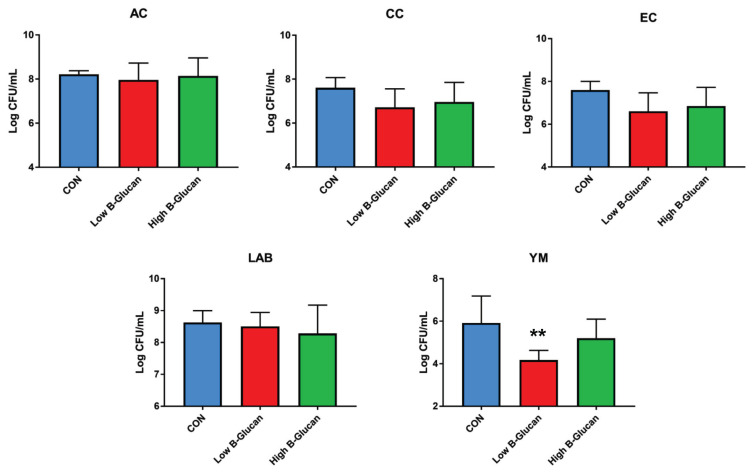
Fecal microbiota according to the addition of β-glucan with vitamin E in weaning pig feed (comparison according to β-glucan; quantitative analysis). Counts were recorded as colony forming units per gram (CFU/g). Highly significant differences in the figure are expressed as **, and differences were considered highly significant at p<0.01. CON, corn-SBM based diet. Low β-glucan meant 0.1% β-glucan and high β-glucan meant 0.2% β-glucan. AC, aerobic count; CC, coliform count; EC, *E.coli*/coliform count; LAB, lactic acid bacteria count; YM, yeast and mold count.

**Figure 3:**
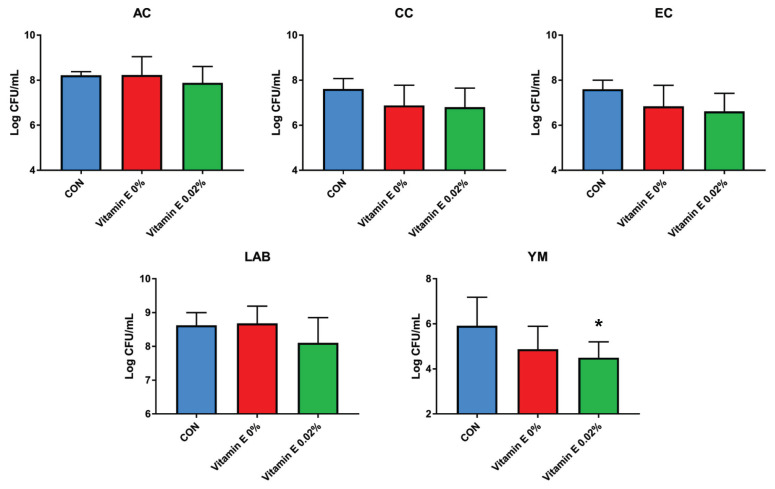
Fecal microbiota according to the addition of β-glucan with vitamin E in weaning pig feed (comparison according to vitamin E; quantitative analysis). Counts were recorded as colony forming units per gram (CFU/g). Significant differences in the figure are expressed as *, and differences were considered significant at p<0.05. CON, corn-SBM based diet. AC, aerobic count; CC, coliform count; EC, *E.coli*/coliform count; LAB, lactic acid bacteria count; YM, yeast and mold count.

**Figure 4:**
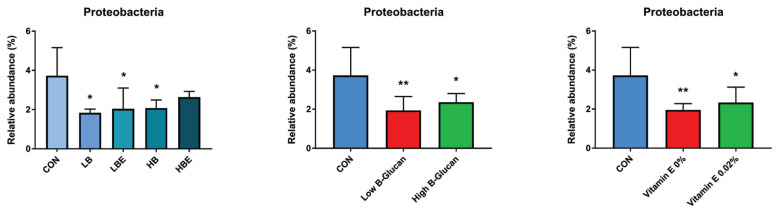
Fecal microbiota according to the addition of β-glucan with vitamin E in weaning pig feed (relative abundance phylum; next generation sequencing analysis). Differences were declared significant at p<0.05 with * marks and highly significant differences were expressed at p<0.01 with ** marks. CON, corn-SBM based diet; LB, corn-SBM based diet+0.1% β-glucan; LBE, corn-SBM based diet+0.1% β-glucan+0.02% vitamin E; HB, corn-SBM based diet+0.2% β-glucan; HBE, corn-SBM based diet+0.2% β-glucan+0.02% vitamin E. Low β-glucan meant 0.1% β-glucan and high β-glucan meant 0.2% β-glucan.

**Figure 5:**
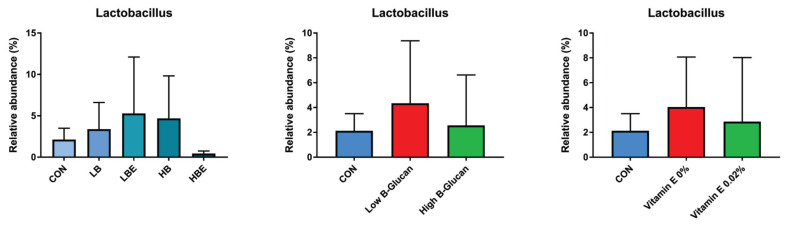
Fecal microbiota according to the addition of β-glucan with vitamin E in weaning pig feed (relative abundance genus; next generation sequencing analysis). CON, corn-SBM based diet; LB, corn-SBM based diet+0.1% β-glucan; LBE, corn-SBM based diet+0.1% β-glucan+0.02% vitamin E; HB, corn-SBM based diet+0.2% β-glucan; HBE, corn-SBM based diet+0.2% β-glucan+0.02% vitamin E. Low β-glucan meant 0.1% β-glucan and high β-glucan meant 0.2% β-glucan.

The effects of BG with VE supplementation in the diet of weaning pigs on fecal microbiota, including aerobic count (AC), coliform count (CC), *E. coli*/coliform count (EC), lactic acid bacteria count, and YM, are shown in Figures 1, 2, 3, 4, and 5. As a result, *Lactobacillus* was decreased in the HBE and YM treatments and was decreased in the LB and LBE treatments compared to the control, as shown in Figure 1 (Diet, p<0.05). Treatments with 0.1% BG showed significantly lower YM compared to that of the control, as shown in Figure 2 (Diet, p<0.01). Treatments with 0.02% VE also showed significantly lower YM compared to that of the control, as shown in Figure 3 (Diet, p<0.05). In addition, the number of Proteobacteria (phylum containing pathogenic microorganisms such as *E. coli*, *Salmonella*, *Shigella*, *etc*.) was significantly lower in the treatments with BG and VE than in the control, as shown in Figure 4 (Diet, p<0.05). According to Figure 5, pigs fed 0.1% BG showed an increasing trend of *Lactobacillus* compared to that of the control.

According to a previous study by Park et al [15], no significant difference was found among treatments in *Lactobacillus* and *Salmonella*, but coliform bacteria decreased linearly in feces as the amount of BG increased in week six when dietary supplementation of BG by level (0%/0.1%/0.2%/0.4%) in the weaning pig diet was compared with the treatment with 0.003% antibiotic Tiamulin (p<0.05). Additionally, *Lactobacillus* and *E. coli* in feces in week two and five were not affected by supplementation with 0.1% each of BG from mulberry leaves and curcuma in the weaning pig feed [18].

In the present experiment, YM decreased when 0.1% BG or 0.02% VE was added to the weaning pig feed. In addition, Proteobacteria decreased compared to the control when 0.1% and 0.2% BG and 0.02% VE were added. Metzler-Zebeli et al [24] reported that supplementing BG could help the composition and metabolic activity of the microbiome in the gastric cavity, cecum, and colon. In another previous study, it was reported that BG, in a mixed form as a grain or concentrate, was easily fermented, decreased the number of intestinal bacteria, and increased the intestinal butyrate concentration of growing pigs [25]. In addition, weaning pigs fed a diet supplemented with BG for two weeks after weaning had reduced susceptibility to enterotoxigenic *E. coli*, a major cause of diarrhea [26]. No significant difference was found in the aerobic bacteria/*E. coli*/coliform bacteria. However, in the current experiment, the decrease in YM and Proteobacteria and the tendency for *Lactobacillus* to increase when supplementing 0.1% BG and 0.02% VE would have benefitted pig health through improvement of the intestinal environment.

### Fecal score

The effects of BG with VE supplementation in the weaning pig diet on fecal score are shown in Table 5. As a result of the experiment, the treatment groups to which BG or VE was added had significantly lower fecal scores than those of the control at week three and six (Diet, p<0.05). Furthermore, treatments with 0.2% BG had significantly lower fecal scores than treatments with 0.1% BG (p<0.05). Treatments with 0.02% VE also significantly showed lower fecal scores than treatments without VE at week three (p<0.05).

In general, diarrhea in weaning pigs occurs over a period of 1 to 2 weeks after weaning due to a change in feed and causes damage to the digestive system. In addition, diarrhea occurs due to a decrease in absorption capacity, which is affected by shortening the length of villi, increasing the depth of crypts, and decreasing the action of digestive enzymes. A decrease in the absorption capacity of the small intestine is associated with the growth of enterotoxic bacteria or a decrease in the fermentation of digestible nutrients in the large intestine, which causes diarrhea in weaning pigs.

According to a previous study by Park et al [15], no significant difference was found among treatments when dietary supplementation of BG by level (0%/0.1%/0.2%/0.4%) in the weaning pig diet was compared with the treatment supplemented with 0.003% of the antibiotic Tiamulin. Lee et al [18] also reported that the fecal score was not affected by supplementation with 0.1% each of BG from mulberry leaves and curcuma in weaning pig feed. On the other hand, the treatment supplemented with 0.0108% BG showed a significantly lower fecal score compared to that of the control and the treatment supplemented with 0.0054% BG when BG by level (0%/0.0054%/0.0108%) was supplemented to weaning pigs experimentally infected with a pathogenic *E. coli* (p<0.05) [27].

In the current experiment, the fecal score was significantly lower when 0.1% or 0.2% BG and 0.02% VE were added to weaning pig feed. Kim et al [27] reported that the fecal score after weaning was low due to the enhancement of the barrier function and immunity of weaning pigs by adding BG. In addition, supplying additional VE is important because VE absorption can be greatly reduced before and after weaning for pigs with diarrhea [23]. Therefore, it was assumed that the fecal score was low because of strengthening the gut integrity of weaning pigs, improving immunity, and reducing oxidative stress through antioxidant effects with supplementation of VE.

Therefore, the results of the present experiment showed that the addition of 0.1% or 0.2% BG and 0.02% VE to weaning pig feed significantly lowered the fecal score.

### Nutrient digestibility

The effects of BG with VE supplementation in the weaning pig diet on nutrient digestibility are shown in Table 6. Supplementing BG and VE did not affect nutrient digestibility and nitrogen retention.

According to the previous study of Lee et al [18], treat ments supplemented with 0.1% each of BG from mulberry leaves and curcuma showed higher digestibility of DM and energy than those of the control over two weeks. Hahn et al [4] conducted an experiment with the addition of BG by level (0%/0.01%/0.02%/0.03%/0.04%) in weaning pig feed. The digestibilities of DM, gross energy, CP, ether extract, Ca, and P increased linearly (p<0.05) as the addition level of BG increased. When dietary supplementation of BG by level (0%/0.1%/0.2%/0.4%) in the weaning pig diet was compared with the treatment with 0.003% of the antibiotic Tiamulin, supplementation of BG linearly increased apparent total tract digestibility of DM and energy during 1–14 and 1–42 days as the amount of BG increased from 0.1% to 0.4% [15]. On the other hand, there was also a study in which the addition of BG in pig feed showed different results from previous studies in terms of nutrient digestibility. The addition of 0.1% BG to growing pig feed had no effect on the digestibility of DM, gross energy, CP, crude ash, or P [28].

In the present study, the addition of BG and VE to wean ing pig feed did not affect nutrient digestibility. This current study did not show an increase in nutrient digestibility, as in previous studies by Hahn et al [4], Lee et al [18], and Park et al [15]. The results also differed from those of a previous study by Brennan and Cleary [29], who reported that the addition of cereal mixed-linked β-(1,3)–(1,4)-d-glucan had a negative effect on the nutrient digestibility and growth performance of pigs.

Further research is needed on the effect of BG obtained from brewer’s yeast used in the current experiment on nutrient digestibility in weaning pigs because the structure, chemical composition, and the effect of BG were different depending on the source type.

In the current experiment, the addition of BG and VE to weaning pig feed had no effect on the nutrient digestibility of weaning pigs.

## CONCLUSION

A significant decrease in yeast and mold count and Proteobacteria and a tendency of increased Lactobacillus compared to the control was shown when 0.1% β-glucan and 0.02% vitamin E were added. The fecal score in weaning pigs was lower in the treatments supplemented with 0.1% or 0.2% β-glucan and 0.02% vitamin E compared to the control. In addition, vitamin E was better supplied to weaning pigs by increasing the concentration of α-tocopherol in the blood of weaning pigs when 0.02% vitamin E was supplemented. Therefore, the addition of 0.1% β-glucan and 0.02% vitamin E to weaning pig feed is thought to have a positive effect on the growth performance of weaning pigs by improving the intestinal microbial composition and reducing the occurrence of diarrhea while efficiently supplying vitamin E.

## Figures and Tables

**Table 1:** Formula and chemical compositions of the experimental diets during weaning phase 1 (0–3 weeks)

Items	Treatment^1)^				

	CON	LB	LBE	HB	HBE
Ingredient (%)					
Expanded corn	39.84	39.65	39.60	39.46	39.42
Corn	10.00	10.00	10.00	10.00	10.00
Soybean meal	34.03	34.06	34.08	34.09	34.10
Soy oil	0.34	0.40	0.41	0.46	0.47
Sweet whey powder	4.00	4.00	4.00	4.00	4.00
Lactose	8.00	8.00	8.00	8.00	8.00
L-Lysine-HCl (78%)	0.26	0.26	0.26	0.26	0.26
DL-methionine (80%)	0.10	0.10	0.10	0.10	0.10
L-threonine (99%)	0.11	0.11	0.11	0.11	0.11
MDCP	1.51	1.51	1.51	1.51	1.51
Limestone	1.26	1.26	1.26	1.26	1.26
Vitamin. Mix^2)^	0.10	0.10	0.10	0.10	0.10
Mineral. Mix^3)^	0.10	0.10	0.10	0.10	0.10
Salt	0.30	0.30	0.30	0.30	0.30
Zinc oxide	0.05	0.05	0.05	0.05	0.05
β-glucan^4)^	0.00	0.10	0.10	0.20	0.20
Vitamin E^4)^	0.00	0.00	0.02	0.00	0.02
Sum	100.00	100.00	100.00	100.00	100.00
Chemical composition (%)^5)^					
ME (kcal/kg)	3,400.00	3,400.00	3,400.00	3,400.00	3,400.00
Crude protein (%)	20.56	20.56	20.56	20.56	20.56
Lysine (%)	1.35	1.35	1.35	1.35	1.35
Methionine (%)	0.39	0.39	0.39	0.39	0.39
Cysteine (%)	0.35	0.35	0.35	0.35	0.35
Threonine (%)	0.79	0.79	0.79	0.79	0.79
Tryptophan (%)	0.22	0.22	0.22	0.22	0.22
Calcium (%)	0.80	0.80	0.80	0.80	0.80
Phosphorus (%)	0.65	0.65	0.65	0.65	0.65

MDCP, mono-dicalcium phosphate; ME, metabolizable energy; SBM, soybean meal

1) CON, corn-SBM based diet; LB, corn-SBM based diet+0.1% β-glucan; LBE, corn-SBM based diet+0.1% β-glucan+0.02% Vitamin E; HB, corn-SBM based diet+0.2% β-glucan; HBE, corn-SBM based diet+0.2% β-glucan+0.02% Vitamin E.

2) Provided the following quantities of vitamins per kg of complete diet: Vitamin A, 11,000 IU; Vitamin D_3_, 920 IU; Vitamin E, 65 IU; Vitamin K_3_, 7.5 mg; Rivoflavin, 8.5 mg; Calcium pantothenic acid, 37 mg; Niacin, 55 mg; D-Biotin, 0.19 mg; Vitamin B_12_, 0.045 mg.

3) Provided the following quantities of minerals per kg of complete diet: Fe, 75 mg; Cu, 32 mg; Mn, 30 mg; I, 0.25 mg; Se, 0.1 mg; Zn, 23 mg.

4) β-glucan and vitamin E products were provided by E&T company (E&T Co, Ltd. Daejeon, South Korea).

5) Calculated value.

**Table 2:** Formula and chemical compositions of the experimental diets during weaning phase 2 (3 to 6 weeks)

Items	Treatment^1)^				

	CON	LB	LBE	HB	HBE
Ingredient (%)					
Expanded corn	30.77	30.59	30.56	30.39	30.36
Corn	30.00	30.00	30.00	30.00	30.00
Soybean meal	29.68	29.70	29.70	29.74	29.74
Soy oil	0.22	0.28	0.29	0.34	0.35
Sweet whey powder	2.00	2.00	2.00	2.00	2.00
Lactose	4.00	4.00	4.00	4.00	4.00
L-Lysine-HCl (78%)	0.23	0.23	0.23	0.23	0.23
DL-methionine (80%)	0.04	0.04	0.04	0.04	0.04
L-threonine (99%)	0.04	0.04	0.04	0.04	0.04
MDCP	1.37	1.37	1.37	1.37	1.37
Limestone	1.12	1.12	1.12	1.12	1.12
Vitamin. Mix^2)^	0.10	0.10	0.10	0.10	0.10
Mineral. Mix^3)^	0.10	0.10	0.10	0.10	0.10
Salt	0.30	0.30	0.30	0.30	0.30
Zinc oxide	0.03	0.03	0.03	0.03	0.03
β-glucan^4)^	0.00	0.10	0.10	0.20	0.20
Vitamin E^4)^	0.00	0.00	0.02	0.00	0.02
Sum	100.00	100.00	100.00	100.00	100.00
Chemical composition (%)^5)^					
ME (kcal/kg)	3,350.00	3,350.00	3,350.00	3,350.00	3,350.00
Crude protein (%)	18.88	18.88	18.88	18.88	18.88
Lysine (%)	1.23	1.23	1.23	1.23	1.23
Methionine (%)	0.36	0.36	0.36	0.36	0.36
Cysteine (%)	0.32	0.32	0.32	0.32	0.32
Threonine (%)	0.73	0.73	0.73	0.73	0.73
Tryptophan (%)	0.20	0.20	0.20	0.20	0.20
Calcium (%)	0.70	0.70	0.70	0.70	0.70
Phosphorus (%)	0.60	0.60	0.60	0.60	0.60

MDCP, mono-dicalcium phosphate; ME, metabolizable energy; SBM, soybean meal

1) CON, corn-SBM based diet; LB, corn-SBM based diet+0.1% β-glucan; LBE, corn-SBM based diet+0.1% β-glucan+0.02% Vitamin E; HB, corn-SBM based diet+0.2% β-glucan; HBE, corn-SBM based diet+0.2% β-glucan+0.02% Vitamin E.

2) Provided the following quantities of vitamins per kg of complete diet: Vitamin A, 11,000 IU; Vitamin D_3_, 920 IU; Vitamin E, 65 IU; Vitamin K_3_, 7.5 mg; Rivoflavin, 8.5 mg; Calcium pantothenic acid, 37 mg; Niacin, 55 mg; D-Biotin, 0.19 mg; Vitamin B_12_, 0.045 mg.

3) Provided the following quantities of minerals per kg of complete diet: Fe, 75 mg; Cu, 32 mg; Mn, 30 mg; I, 0.25 mg; Se, 0.1 mg; Zn, 23 mg.

4) β-glucan and vitamin E products were provided by E&T company (E&T Co, Ltd. Daejeon, Korea).

5) Calculated value.

**Table 3:** Effects of β-glucan with vitamin E supplementation on growth performance in weaning pigs

Items	Treatment^1)^					SEM	p-value			
	
	CON	LB	LBE	HB	HBE		Diet	BG	VE	BG×VE
Body weight (kg)										
Initial	--------------------------------------- 7.64 ---------------------------------------					0.148	-	-	-	-
Week 3	10.44	10.72	10.29	10.74	10.75	0.121	0.57	0.42	0.47	0.46
Week 6	18.76	18.90	20.38	20.32	20.93	0.231	<0.01	<0.01	<0.01	0.15
Average daily gain (g)										
0 to 3 weeks	133.59	146.78	126.35	147.62	148.04	5.347	0.54	0.37	0.43	0.41
3 to 6 weeks	395.98	389.11	480.07	456.62	484.58	12.259	0.02	0.09	<0.01	0.13
0 to 6 weeks	264.79	268.76	303.21	301.83	316.32	6.291	0.03	0.08	0.06	0.42
Average daily feed intake (g)										
0 to 3 weeks	254.04	268.78	239.40	267.81	257.45	7.930	0.84	0.65	0.31	0.62
3 to 6 weeks	624.98	636.67	652.55	703.58	705.20	10.559	0.02	<0.01	0.63	0.69
0 to 6 weeks	439.51	452.73	445.96	485.69	481.33	7.588	0.14	0.04	0.72	0.94
Gain:feed ratio (G:F ratio)										
0 to 3 weeks	0.531	0.554	0.532	0.553	0.593	0.025	0.69	0.62	0.89	0.65
3 to 6 weeks	0.635	0.611	0.736	0.650	0.690	0.016	0.32	0.90	0.02	0.20
0 to 6 weeks	0.606	0.594	0.679	0.622	0.663	0.015	0.38	0.86	0.08	0.52

SEM, standard error of the mean; BG, β-glucan; VE, vitamin E; SBM, soybean meal.

1) CON, corn-SBM based diet; LB, corn-SBM based diet+0.1% β-glucan; LBE, corn-SBM based diet+0.1% β-glucan+0.02% Vitamin E; HB, corn-SBM based diet+0.2% β-glucan; HBE, corn-SBM based diet+0.2% β-glucan+0.02% Vitamin E.

**Table 4:** Effects of β-glucan with vitamin E supplementation on blood profiles and immune response in weaning pigs

Items	Treatment^1)^					SEM	p-value			
	
	CON	LB	LBE	HB	HBE		Diet	BG	VE	BG×VE
Vitamin E (μmol/L)										
Initial	----------------------------------------- 8.90 -----------------------------------------					-	-	-	-	-
Week 3	2.00	2.35	2.95	2.13	2.73	0.271	0.51	0.65	0.08	1.00
Week 6	2.10	3.67	3.27	3.60	3.93	0.400	0.74	0.49	0.91	0.83
Selenium (μ/L)										
Initial	----------------------------------------- 99.00 -----------------------------------------					-	-	-	-	-
Week 3	125.00	105.67	127.67	130.67	120.67	4.064	0.35	0.51	0.51	0.10
Week 6	158.00	167.34	175.33	125.00	148.67	7.002	0.17	0.08	0.28	0.58
TNF-α (μmol/L)										
Initial	----------------------------------------- 0.47 -----------------------------------------					-	-	-	-	-
Week 3	0.18	0.20	0.21	0.20	0.23	0.201	0.99	0.99	0.90	0.95
Week 6	0.04	0.16	0.07	0.02	1.92	0.350	0.38	0.56	0.26	0.22
IL-6 (μmol/L)										
Initial	----------------------------------------- 0.91 -----------------------------------------					-	-	-	-	-
Week 3	1.01	1.72	1.26	0.15	1.55	0.307	0.58	0.68	0.52	0.22
Week 6	0.18	0.50	0.13	0.17	0.29	0.078	0.62	0.71	0.49	0.21
Lymphocyte (%)										
Initial	----------------------------------------- 54.30 -----------------------------------------					-	-	-	-	-
Week 3	51.13	43.97	44.70	44.47	45.13	1.512	0.61	0.32	0.85	0.99
Week 6	54.80	50.07	52.53	48.65	53.80	1.769	0.87	0.52	0.34	0.73

SEM, standard error of the mean; BG, β-glucan; VE, vitamin E; TNF-α, tumor necrosis factor-α; IL, interleukin; SBM, soybean meal.

1) CON, corn-SBM based diet; LB, corn-SBM based diet+0.1% β-glucan; LBE, corn-SBM based diet+0.1% β-glucan+0.02% Vitamin E; HB, corn-SBM based diet+0.2% β-glucan; HBE, corn-SBM based diet+0.2% β-glucan+0.02% Vitamin E.

**Table 5:** Effects of β-glucan with vitamin E supplementation on the fecal score in weaning pigs

Items	Treatment^1)^					SEM	p-value			
	
	CON	LB	LBE	HB	HBE		Diet	BG	VE	BG×VE
Fecal score^2)^										
Week 3	1.09	0.89	0.69	0.79	0.65	0.048	<0.01	0.04	0.04	0.69
Week 6	0.80	0.58	0.49	0.66	0.65	0.042	0.04	0.13	0.55	0.65

SEM, standard error of the mean; BG, β-glucan; VE, vitamin E; SBM, soybean meal

1) CON, corn-SBM based diet; LB, corn-SBM based diet+0.1% β-glucan; LBE, corn-SBM based diet+0.1% β-glucan+0.02% Vitamin E; HB, corn-SBM based diet+0.2% β-glucan; HBE, corn-SBM based diet+0.2% β-glucan+0.02% Vitamin E.

2) Fecal score: 0, normal feces; 1, moist feces; 2, mild diarrhea; 3, watery diarrhea.

**Table 6:** Effects of β-glucan with vitamin E supplementation on nutrient digestibility in weaning pigs^1)^

Items	Treatment^2)^					SEM	p-value			
	
	CON	LB	LBE	HB	HBE		Diet	BG	VE	BG×VE
Nutrient digestibility (%)										
Dry matter	91.41	91.53	91.83	91.55	92.25	0.74	0.85	0.90	0.79	0.91
Crude protein	90.10	88.62	89.51	90.62	90.58	0.99	0.92	0.53	0.86	0.85
Crude ash	72.52	71.35	73.69	72.39	73.78	2.29	0.96	0.92	0.75	0.93
Crude fat	80.99	82.33	81.17	80.01	81.45	1.97	0.96	0.84	0.97	0.79
N-retention (g/d)										
N-intake	5.13	5.13	5.14	5.13	5.16	-	-	-	-	-
N-feces	0.51	0.58	0.54	0.48	0.49	0.25	0.92	0.54	0.87	0.84
N-urine	2.35	2.26	2.12	2.24	2.10	0.19	0.18	0.83	0.10	0.97
N-retention^3)^	2.27	2.28	2.48	2.40	2.57	0.35	0.39	0.52	0.28	0.93

SEM, standard error of the mean; BG, β-glucan; VE, vitamin E; SBM, soybean meal.

1) A total of 15 barrows (initial body weight, 12.48±0.37 kg) were used.

2) CON, corn-SBM based diet; LB, corn-SBM based diet+0.1% β-glucan; LBE, corn-SBM based diet+0.1% β-glucan+0.02% Vitamin E; HB, corn-SBM based diet+0.2% β-glucan; HBE, corn-SBM based diet+0.2% β-glucan+0.02% Vitamin E.

3) N-retention = N intake (g)–fecal N (g)–urinary N (g).
